# Successful elimination of premature ventricular contractions by ablation of origin and preferential pathway

**DOI:** 10.1002/ccr3.1261

**Published:** 2017-11-23

**Authors:** Kyoichiro Yazaki, Kenji Enta, Masahiro Watarai, Mitsuru Kahata, Asako Kumagai, Koji Inoue, Hiroshi Koganei, Masato Otsuka, Yasuhiro Ishii

**Affiliations:** ^1^ Department of Cardiology Cardiovascular Center Ogikubo Hospital Tokyo Japan

**Keywords:** Catheter ablation, preferential pathway, premature ventricular contraction, prepotential, stimulus–QRS latency

## Abstract

However, the common strategy for eliminating premature ventricular contractions (PVCs) is to explore the exit site and ablate, which may be difficult in some cases. The origin and the preferential pathway, an insulated pathway connected to the exit, may also become targets for eliminating PVCs.

## Introduction

Premature ventricular contractions (PVCs) originating from the aortic cusps account for approximately 15% of all idiopathic ventricular arrhythmias 1. The strategy for eliminating these arrhythmias is based mainly on the following two concepts: (1) ablation at the exit site and (2) ablation of the origin or the preferential pathway, an insulated connection between origin and exit. The latter method is relatively more uncommon than the former, and it is difficult to prove the entity of them 2. We present a case of successful catheter ablation of outflow tract PVCs by modification of the origin and preferential pathway.

## Case Report

**Figure 1:**
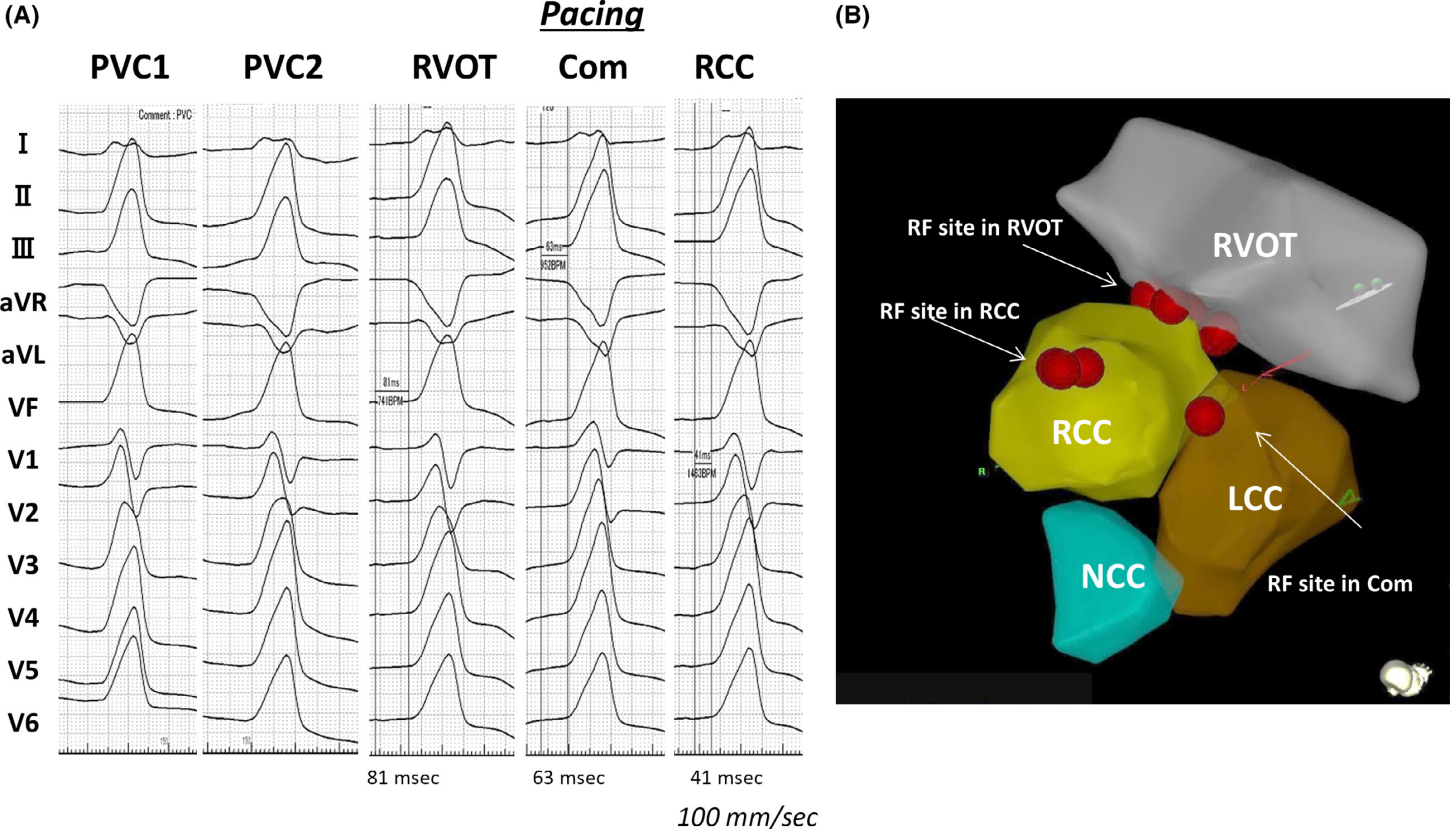
(A) A 12‐lead electrocardiogram during PVC1, PVC2, and pacing in the right ventricular outflow tract, RCC–LCC commissure, and RCC. (B) Three‐dimensional electroanatomical mapping and the radiofrequency application points. PVC, premature ventricular contraction; RCC, right coronary cusp; LCC, left coronary cusp; Com, RCC–LCC commissure; RF, radiofrequency; RVOT, right ventricular outflow tract.

A 57‐year‐old woman who complained of frequent palpitations was referred to our clinic. The transthoracic echocardiogram revealed no structural heart disease, and a 24‐h Holter monitoring detected PVCs occurring approximately 25,000 times/day, with left bundle branch block, inferior axis, and precordial transition in lead V_2_. The PVCs all had the same coupling interval of 440 msec and showed two different morphologies (PVC1, PVC2), with PVC1 having a deeper S wave in lead V_2_ (Fig. 1A). We had already failed to ablate them from the right ventricular outflow tract (RVOT) with a nonirrigated‐tip catheter 6 months previously. We scheduled a second session to target these two PVCs.

**Figure 2:**
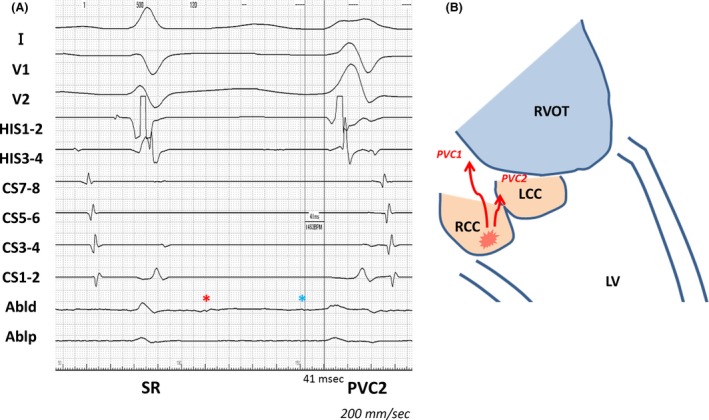
(A) Intracardiac electrogram showing a local potential preceding QRS onset (blue asterisk) and a late potential during sinus rhythm (red asterisk) at the RCC. The interval between the second component of the local potential and the QRS onset was 41 msec during PVC2. (B) Diagram showing the origin and route of the preferential pathways that we postulated (red line). Abld, distal electrode of ablation catheter; Ablp, proximal electrode of ablation catheter; CS, coronary sinus electrode; HIS, His bundle electrode; LCC, left coronary cusp; LV, left ventricle; PVC, premature ventricular contraction; RCC, right coronary cusp; RVOT, right ventricular outflow tract; SR, sinus rhythm.

The electroanatomical mapping (EAM) and subsequent radiofrequency application (RFA) were performed using a three‐dimensional EAM system (Carto3^®^; Biosense Webster, Diamond Bar, CA) with a 3.5‐mm open‐irrigated‐tip catheter (Thermocool SmartTouch^®^ SF; Biosense Webster). An ultrasound imaging system (CARTO SOUND™; Biosense Webster) was utilized for reconstruction of the geometry of the RVOT and aortic cusp, providing extra information about the structural relationship between them (Fig. 1B). First, we performed pace mapping at the mid‐septum in the RVOT, which produced a QRS morphology similar to PVC1 with a stimulus–QRS latency of 81 msec (Fig. 1A); there was no other site where pace mapping produced a QRS complex identical to that of the clinical PVC. In addition, we could not detect the local potential preceding the QRS onset during PVCs or any late potentials during sinus rhythm. Nevertheless, after RFA at this site, PVC1 disappeared; PVC2, however, was still present. Therefore, we next performed mapping at the aortic cusp. Pace mapping was applied at the RCC or RCC–left coronary cusp (LCC) commissure. Pacing with an output of 10 V at 0.5 msec at the RCC–LCC commissure produced a QRS morphology quite similar to that of PVC2, with a stimulus–QRS latency of 63 msec (Fig. 1A). In contrast, pacing at the RCC with the same settings produced a QRS morphology identical to that of PVC2, with a stimulus–QRS latency of 41 msec (Fig. 1A). Furthermore, we were able to record a local potential preceding the QRS onset during PVC2, as well as late potentials during sinus rhythm that consisted of two components in the RCC (Fig. 2A). Several RFAs at the RCC–LCC commissure failed to eliminate PVCs, but subsequent RFAs at the RCC were successful, using a power of 30 W and up to 42°C for 90 sec, without any recurrence of either PVC1 or PVC2. The patient was fully informed about the diagnostic and therapeutic procedures and gave her consent.

## Discussion

We have described a case of successful catheter ablation of two types of PVCs using RFA in the RVOT and aortic cusp.

The RCC is a relatively rare site of PVC origin compared with the RVOT or LCC 1. An early local potential preceding the QRS onset is one of the indicators for successful ablation 3, 4. In some cases, high output is required to perform pace mapping, suggesting a distant origin, or the presence of a preferential pathway. Therefore, it has been speculated that pacing from the aortic cusp might directly capture the origin or preferential pathway, representing it as stimulus–QRS latency 2. In the case of PVC1, successful pace mapping was achieved in the RVOT, where PVC1 was eliminated by RFA using an open‐irrigated‐tip catheter; this suggested that the breakout site was located in the RVOT. In the case of PVC2, an acceptable pace map for PVC2 was recorded at the RCC–LCC commissure, possibly suggesting that it might be the breakout site of PVC2. However, the successful ablation site was in the RCC, where excellent pace mapping was available. In addition, late potentials during sinus rhythm and prepotentials during PVC2 were successfully recorded in the RCC, strongly suggesting that the PVC's origin was likely to be located within the RCC. Interestingly, the second component of the local potential preceding the QRS onset by 41 msec during PVC2 was identical to the stimulus–QRS latency during pacing from the RCC, also supporting the above hypothesis. Furthermore, PVC1 and PVC2 had the same coupling interval and the only electrocardiographic difference was in the depth of the S wave in V_2_. From these findings, we speculated that both PVCs shared the same origin in the RCC, but had different exits (Fig. 2B). Shirai et al. reported a similar case 5, clearly describing the entity of a bifurcated preferential pathway and determination of the exit site by pace mapping and the analysis of local potentials. In our case, too, we were able to clarify the existence of the origin and the preferential pathway and treat it using an open‐irrigated‐tip catheter, resulting in complete elimination of the PVC. However, such latency during pace mapping in the RVOT is very rare. We speculated that the pacing in the RVOT had directly captured the preferential pathway or that the pacing site might have been above the pulmonary valve, as a previous report suggested 6.

This case provides us with two significant lessons: (1) stimulus–QRS latency implies the presence of a distant origin and an associated preferential pathway, which can be an indicator for successful elimination of PVCs by RFA and (2) late potentials during sinus rhythm or prepotentials during PVCs indicate an insulated pathway that may also become a target for RFA.

## Conclusion

There are several indicators of the origin and preferential pathway of outflow tract PVCs, which can become a target for the elimination of the arrhythmia. We believe that this case report has important clinical implications when we consider the therapeutic approach to outflow tract PVCs.

## Conflict of Interest

None declared.

## Authorship

KY: collected and interpreted the clinical data and performed drafting, editing, and revision. KY, KE, MW, MK, AK, KI, HK, MO, and YI: gave final approval.
